# Books Reviewed

**Published:** 1865-08

**Authors:** 


### Books Reviewed.

Renewal of Life—Lectures chiefly Clinical. By Thomas King Chambers, M. D., Honorary Physician to H. R. H. Prince of Wales; Physician to St. Mary's and the Lock Hospitals. From the third London edition. Philadelphia: Lindsay & Blakiston, 1865.

This volume comprises fifty-two lectures, chiefly clinical, on practical medicine. Three of which were delivered at the College of Physicians, and the rest at St. Mary's Hospital, by the author, in his capacity as teacher of Clinical Medicine in that institution, upon the following subjects:—Death and Life, Disease and Cure, Formation of Mucus and Pus, Typh-Fever, Small-Pox, Rheumatic Fever, Gonorrhœal Rheumatism, Pericarditis, Pleurisy, Hydrothorax, Acute Laryngitis, Capillary Catarrh, Pneumonia, Emphysema of Lungs, Pulmonary Consumption, Thoracic Aneurism, Disease of Heart, Purpura, Anæmia, Prominent of Eyeballs, Atrophy of Muscles, Chorea, Epilepsy, Hysteria, Spinal Paralysis, Sciatica, Albuminuria, Ascites, Diabetes, Mortification, Importance of the Digestive Organs in Therapeutics, Indigestion in General, Slow Digestion and Acidity, Pain in the Stomach, Eructation and Vomiting, Diarrhœa, Costiveness and Constipation, Dietetics, Corpulence, On Pepsine, On Alcohol, On Blood Letting, Answer to Objections.

We have copied the headings of each lecture in full, in order that our readers may judge of the character of the work. The two former editions of this work were named "The Renewal of Life," intending thereby to intimate that the main point for the physician's consideration in disease is the deficiency of vital action, and that all successful medical treatment is the renewal of that vital action.

The author regrets that the words were found strangely "open to misrepresentations" by several of the literary men engaged in reviewing the work, which has led to the unusual course of leaving out a great part of the title; but the publishers of this, the American edition, have thought it advisable to retain, in part, the title of the two former editions. It is written in the finest style of our language, which makes its perusal doubly attractive; and we know of no medical work we would more cheerfully recommend to the profession. The chapter upon Alcohol, is made very valuable by the introduction of some well conducted experiments to show its effects upon the healthy subject. After giving the results in tabular form it says:

"On the whole, we may conclude that the effect of continued small doses of alcohol, is to diminish the vital metamorphosis, to make it irregular, and to induce, in healthy people, the necessity for crisis of evacuation. Its first action is upon the stomach, enabling more food to be digested, and increasing vitality; but if advantage is not taken of this first action, its secondary effect is a diminution of vital functions in general and of digestion among their number."

In his farewell chapter he says:—"I would call upon all to remember what a high matter it is that we take upon ourselves to handle. Man's life!—that which makes him God's viceroy on earth; for divorced dust and spirit cease to hold that lofty post. To aid us in our duty we are endowed with dominion over not only brute matter, which we can number and weigh, but over those unseen forces which our reason makes known to us; heat, electricity, vitality, and may be other yet nameless 'powers of the Lord.' Our business is to use them to lengthen and lighten man's earthly trial. He, in our profession, who is first in the scale of humanity is the first and best physician."

---

Hand-Book of Skin Diseases for Students and Practitioners. By Thomas Hillier, M. D., London. Philadelphia: Blanchard & Lea, 1865.

This is a work of 350 pages. The author, in his capacity as Physician to the Skin Department of University College Hospital, has had a large field of experience in the diseases.

In most of our medical colleges the advantages for clinical instruction in this class of diseases is limited, consequently a book of this kind is of inestimable value to the student. During the last few years our knowledge of skin diseases has been considerably extended, and the diagnosis of many skin diseases made positive by the application of the microscope. In this work a few original wood engravings, taken from cases under the author's care, have been introduced to illustrate the microscopical appearances presented by the hair and cuticle when effected by vegetable growth. In the treatment complexity is avoided, by not recording a number of nearly useless remedies, and by stating the principles on which treatment should be based. In re-printing this volume, Neligan's "Atlas of Cutaneous Diseases" is referred to, where the disease is represented in it.

This book is written tastefully, and will give universal satisfaction. We recommend it to the profession as one of the very best.

---

Contributions to Practical Surgery. By Wm. H. Van Buren, M. D., Professor of Anatomy University of New York; formerly one of the Surgeons of the New York Hospital, Bellevule Hospital, and Vice President of the New York Academy of Medicine; consulting Surgeon to St. Vincent's Hospital, and the Woman's Hospital; member of the Pathological Society and the Medical and Surgical Society of New York; member of the U. S. Sanitary Commission, etc., etc. Philadelphia: J. B, Lippincott & Co., 1865.

The work before us consists of a collection of a large number of surgical cases, with their diagnosis, treatment, etc., selected from the professional periodicals in which they were originally published, with the hope that they may be of service to those interested in the pursuit of practical surgery.

If a larger number of our professional friends, who have extensive opportunities for witnessing the results of a large medical and surgical practice, would follow the praiseworthy example of Dr. Van Buren, and afford the profession the result of their actual practice, for a number of years, it would be of inestimable value to the medical man, and especially to those whose opportunities to a certain extent are limited.

All of the cases recorded are of the most interesting and instructive nature, as well as the most important in a surgical point of view. We think this work will afford pleasure and satisfaction to all who read it attentively; it is printed in pamphlet form, and comprises over two hundred pages.

